# The *Infectious Medicine* is becoming a globally high-level scientific journal

**DOI:** 10.1016/j.imj.2024.100091

**Published:** 2024-02-17

**Authors:** Xue-Jie Yu

**Affiliations:** School of Public Health, Wuhan University, Wuhan 430071, China

“Humans are going through an era of epidemics and pandemics”. Escaping the “Pandemic Era” will require transformative changes and no less than a fundamental reassessment of our relationship with nature [1]. As a result, “One Health”, an integrated, unified strategy to balance and improve the health of humans, animals, and the environment was created. The goal of the *Infectious Medicine* is to develop into a comprehensive One Health platform for studying zoonotic and human infectious diseases.

In 2023, the *Infectious Medicine* received a total of 195 manuscripts and finally 42 were published. Among the published papers, the majority were original articles (23), accounting for 80%, followed by “review” and “case report” (11 and 8 respectively). The journal's submissions were from countries around the world including Africa, Asia, Europe, Oceania, North America, and South America. The submission rate of other places outside China reached 78%. The speed of review process has been increasing annually, with a median time from submission to first desk decision of 2 days, from submission to first standard decision of 41 days, and from submission to final decision of 62 days in 2023.

The journal has 4 special issues: Viral genomes in disease surveillance and prevention, Late effect of COVID-19, Vector-borne diseases, and The discovery of molecular targets for tropical infectious disease diagnosis. Among these special issues, “Vector-borne diseases” received the most submissions, but the quality of papers was strictly controlled and the acceptance rate for this special issue was only 20%. Of the published articles, 26 have been cited, the highest cited article has more than 40 citations which addressed the etiology, clinical manifestations, distribution, diagnosis, contact management, risk management and prevention of monkey pox [2]. The *Infectious Medicine* was focusing on the research of the latest infectious diseases around the world. In addition to citations, the published articles have been downloaded a total of 96,100 times, with 18 of the 42 articles being downloaded more than 1000 times. The most downloaded article (16,000 times) was about oral lesions and immune mechanisms after COVID-19 vaccination, submitted by the University of Medical Sciences of Mazandaran in Iran [3]. The United States was the region with the highest number of downloads, with over 50,000 downloads. It demonstrated that the *Infectious Medicine* was serving the field of infectious diseases worldwide and the *Infectious Medicine* is increasingly popular and read by medical and public health workers and researchers around the world.

The *Infectious Medicine* has hosted 8 academic conferences on infection and control, pathogen detection, drug-resistant bacterium prevention and treatment, zoonotic disease prevention and treatment in the time period. The *Infectious Medicine* publishes full articles, short report (case reports), and reviews from all fields of infectious diseases, including the prevention and control of infectious diseases, diagnosis and treatment of infectious diseases, reports on the cross-disciplinary research of emerging infectious diseases and zoonotic diseases. Articles must be innovative, important, or instructive in the field.

The *Infectious Medicine* has been indexed by Scopus, CABI, DOAJ, and PubMed with a citing score of 1.3 in 2023. The journal is being acknowledged and accepted by scientists worldwide and is becoming a high-level journal on a global scale.

## Funding

None.

## Acknowledgments

None.

## Declaration of competing interest

The author declared no potential conflicts of interest with respect to the research, authorship, and/or publication of this article.

## Data available statement

None.

## Ethics statement

None.

## Informed consent

None.
